# Preliminary evidence of safety and tolerability of atezolizumab plus bevacizumab in patients with hepatocellular carcinoma and Child‐Pugh A and B cirrhosis: A real‐world study

**DOI:** 10.1002/hep.32468

**Published:** 2022-04-08

**Authors:** Antonio D’Alessio, Claudia Angela Maria Fulgenzi, Naoshi Nishida, Martin Schönlein, Johann von Felden, Kornelius Schulze, Henning Wege, Vincent E. Gaillard, Anwaar Saeed, Brooke Wietharn, Hannah Hildebrand, Linda Wu, Celina Ang, Thomas U. Marron, Arndt Weinmann, Peter R. Galle, Dominik Bettinger, Bertram Bengsch, Arndt Vogel, Lorenz Balcar, Bernhard Scheiner, Pei‐Chang Lee, Yi‐Hsiang Huang, Suneetha Amara, Mahvish Muzaffar, Abdul Rafeh Naqash, Antonella Cammarota, Nicola Personeni, Tiziana Pressiani, Rohini Sharma, Matthias Pinter, Alessio Cortellini, Masatoshi Kudo, Lorenza Rimassa, David J. Pinato

**Affiliations:** ^1^ Department of Surgery & Cancer Imperial College London Hammersmith Hospital London UK; ^2^ 437807 Department of Biomedical Sciences Humanitas University Pieve Emanuele, Milan Italy; ^3^ Division of Medical Oncology Policlinico Universitario Campus Bio‐Medico Rome Italy; ^4^ Department of Gastroenterology and Hepatology Kindai University Faculty of Medicine Osaka Japan; ^5^ 37734 Department of Oncology, Hematology and Bone Marrow Transplantation with Section of Pneumology University Medical Center Hamburg‐Eppendorf Hamburg Germany; ^6^ 37734 Department of Medicine University Medical Center Hamburg‐Eppendorf Hamburg Germany; ^7^ F. Hoffmann‐La Roche Ltd. Basel Switzerland; ^8^ Division of Medical Oncology Department of Medicine Kansas University Cancer Center Kansas City Kansas USA; ^9^ Division of Hematology/Oncology Department of Medicine Tisch Cancer Institute Mount Sinai Hospital New York New York USA; ^10^ University Medical Center Mainz Mainz Germany; ^11^ Department of Medicine II (Gastroenterology, Hepatology, Endocrinology and Infectious Diseases) Faculty of Medicine Freiburg University Medical Center University of Freiburg Freiburg Germany; ^12^ University of Freiburg Signalling Research Centres BIOSS and CIBSS Freiburg Germany; ^13^ German Cancer Consortium (DKTK), partner site Freiburg Germany; ^14^ Hannover Medical School Hannover Germany; ^15^ 27271 Division of Gastroenterology & Hepatology Department of Internal Medicine III Medical University of Vienna Vienna Austria; ^16^ Division of Gastroenterology and Hepatology Department of Medicine Taipei Veterans General Hospital Taipei Taiwan; ^17^ Institute of Clinical Medicine School of Medicine National Yang Ming Chiao Tung University Taipei Taiwan; ^18^ 3627 Division of Hematology/Oncology East Carolina University Greenville North Carolina USA; ^19^ Medical Oncology/TSET Phase 1 Program Stephenson Cancer Center University of Oklahoma Norman Oklahoma USA; ^20^ Medical Oncology and Hematology Unit Humanitas Cancer Center IRCCS Humanitas Research Hospital Rozzano Milan Italy; ^21^ Division of Oncology Department of Translational Medicine University of Piemonte Orientale Novara Italy

## Abstract

**Background and Aims:**

Atezolizumab plus bevacizumab (AtezoBev) is the standard of care for first‐line treatment of unresectable HCC. No evidence exists as to its use in routine clinical practice in patients with impaired liver function.

**Approach and Results:**

In 216 patients with HCC who were consecutively treated with AtezoBev across 11 tertiary centers, we retrospectively evaluated treatment‐related adverse events (trAEs) graded (G) according to Common Terminology Criteria for Adverse Events v5.0, including in the analysis all patients treated according to label (*n* = 202, 94%). We also assessed overall survival (OS), progression‐free survival (PFS), overall response (ORR), and disease control rates (DCR) defined by Response Evaluation Criteria in Solid Tumors v1.1. Disease was mostly secondary to viral hepatitis, namely hepatitis C (*n* = 72; 36%) and hepatitis B infection (*n* = 35, 17%). Liver function was graded as Child‐Pugh (CP)‐A in 154 patients (76%) and CP‐B in 48 (24%). Any grade trAEs were reported by 143 patients (71%), of which 53 (26%) were G3 and 3 (2%) G4. Compared with CP‐A, patients with CP‐B showed comparable rates of trAEs. Presence and grade of varices at pretreatment esophagogastroduodenoscopy did not correlate with bleeding events. After a median follow‐up of 9.0 months (95% CI, 7.8–10.1), median OS was 14.9 months (95% CI, 13.6–16.3), whereas median PFS was 6.8 months (95% CI, 5.2–8.5). ORR and DCR were respectively 25% and 73%, with no difference across CP classes.

**Conclusions:**

This study confirms reproducible safety and efficacy of AtezoBev in routine practice. Patients with CP‐B reported similar tolerability compared with CP‐A, warranting prospective evaluation of AtezoBev in this treatment‐deprived population.

## Graphical abstract

In this retrospective study on a real‐life cohort of 216 patients treated with Atezolizumab plus Bevacizumab, the combination did not show any unexpected safety signals. Treatment‐related adverse events were comparable across Child‐Pugh (CP) classes. CP‐B patients achieved similar response rates to CP‐A patients, despite inferior survival outcomes.

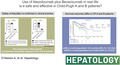

**Abbreviations**
AE: adverse event
ALBI: albumin‐bilirubin
BCLC: Barcelona Clinic Liver Cancer
CP: Child‐Pugh
CR: complete response
DCR: disease control rate
ECOG‐PS: Eastern Cooperative Oncology Group performance status
EGD: esophagogastroduodenoscopy
EHS: extrahepatic spread
GI: gastrointestinal
IQR: interquartile ratio
mAb: monoclonal antibody
mOS: median overall survival
mPFS: median progression‐free survival
NIHR: National Institute for Health Research
ORR: overall response rate
OS: overall survival
PFS: progression‐free survival
PR: partial response
RECIST: Response Evaluation Criteria in Solid Tumors
SD: stable disease
trAE: treatment‐related AE
TTP: time‐to‐progression

## INTRODUCTION

HCC is the cause of over 830,000 annual deaths worldwide, being expected to affect more than one million individuals by 2025.^[^ ^1^, ^2^ ^]^ Since 2008, sorafenib has represented the mainstay of treatment for advanced HCC,^[^ ^3^, ^4^ ^]^ until 2019, when lenvatinib was proven noninferior to sorafenib in the phase III REFLECT trial.^[^ ^5^ ^]^ The treatment landscape of advanced HCC has ever since been profoundly revolutionized by the approval of atezolizumab, an antiprogrammed death ligand 1 monoclonal antibody (mAb), plus bevacizumab, an anti‐VEGF mAb, for first‐line treatment of unresectable or metastatic HCC in the year 2020. IMbrave150^[^ ^6^ ^]^ is the first study to demonstrate superiority of combination immunotherapy over sorafenib as measured by overall survival (OS) and progression‐free survival (PFS), leading to the establishment of a novel global standard of care in unresectable HCC. After an updated median follow‐up of 15.6 months,^[^ ^7^ ^]^ atezolizumab and bevacizumab therapy was shown to extend the median OS (mOS) to 19.2 versus 13.4 months in the sorafenib arm (HR, 0.66; 95% CI, 0.52–0.85; *p* = 0.0009), a finding mirrored by the significant extension of median PFS (mPFS) to 6.9 months versus 4.3 months (HR, 0.65; 95% CI, 0.53–0.81; *p* = 0.0001). The updated overall response rate (ORR) evaluated by independent review per Response Evaluation Criteria in Solid Tumors (RECIST) criteria v1.1^[^ ^8^ ^]^ was 29.8% (95% CI, 24.8–35.0) with atezolizumab plus bevacizumab versus 11.3% (95% CI, 6.9–17.3) with sorafenib, with 7.7% patients treated with the combination achieving a complete response (CR).

Despite having achieved the longest mOS ever reported in clinical trials for advanced HCC, clinical outcomes from the combined use of atezolizumab and bevacizumab in routine clinical practice have not been described in well‐designed multicenter studies.^[^ ^9^ ^]^ As therapeutic options for advanced HCC expand,^[^ ^10^, ^11^ ^]^ it is important to evaluate how candidacy for combination immunotherapy is assessed outside clinical trials, especially given the adverse event (AE) profile of atezolizumab and bevacizumab, which includes immune‐related pathology as well as potential risk of bleeding. In addition, although initial evidence suggests that PD‐1 monotherapy can be safely administered in patients with Child‐Pugh (CP) B liver dysfunction,^[^ ^12^, ^13^, ^14^ ^]^ there is no evidence to confirm safety and efficacy of the atezolizumab and bevacizumab combination in patients with unresectable HCC outside strict CP‐A criteria.

To address these questions, we analyzed the data of patients treated with atezolizumab plus bevacizumab extracted from a global multicenter consortium of patients treated with different immunotherapy regimens that we have prospectively maintained since 2017.^[^ ^13^, ^15^, ^16^, ^17^ ^]^ Although a number of local consortia are evaluating efficacy and tolerability of atezolizumab plus bevacizumab in routine clinical practice,^[^ ^18^, ^19^ ^]^ we conducted a retrospective international analysis focused on describing safety and tolerability in patients with varying degree of liver dysfunction treated with atezolizumab and bevacizumab.

## PATIENTS AND METHODS

### Study design and participants

We conducted a multicenter retrospective study in patients with unresectable or metastatic HCC treated with atezolizumab plus bevacizumab as part of routine clinical care. Patients were treated in 11 tertiary referral centers in Germany (*n* = 55), United States (*n* = 55), Japan (*n* = 51), Austria (*n* = 14), United Kingdom (*n* = 17), Italy (*n* = 12), and Taiwan (*n* = 12) from January 2019 to January 2022. All patients were at least 18 years old, had a histological or radiological diagnosis of HCC according to the American Association for the Study of Liver Diseases criteria,^[^ ^20^ ^]^ and were diagnosed with advanced disease, defined according to the Barcelona Clinic Liver Cancer (BCLC) criteria.

### Treatment administration and outcome measures

Atezolizumab and bevacizumab were administered according to schedule of the IMbrave150 protocol at the following doses: atezolizumab 1200 mg plus bevacizumab 15 mg/kg intravenously every 3 weeks. Treatment was administered following a multidisciplinary assessment and according to the local practice of each participating institution. Toxicity management, including dose modifications, was carried out in accordance with the summary of product characteristics (SmPC) for the two agents. Treatment was continued until disease progression or unacceptable toxicity. Data regarding patients’ demographics and clinical status were collected retrospectively and prospectively maintained and updated at each participating site. We included in the safety and efficacy analysis all patients receiving at least one dose of atezolizumab plus bevacizumab in first line according to the IMbrave150 indication,^[^ ^6^ ^]^ and we excluded patients with prior lines of systemic treatment. We assessed response and survival as an exploratory endpoint. Radiological response to treatment was evaluated per RECIST criteria v1.1 on CT or MRI, performed every 9–12 weeks as part of periodic restaging. AEs were assessed at every contact with the patient and were graded according to the National Cancer Institute Common Terminology Criteria for Adverse Events (CTCAE) v5.0. Only AEs deemed to be treatment‐related were collected, and attribution of causality to either drug was based on the published toxicity profile of atezolizumab and bevacizumab, the assessment of treating physicians at each center, and the judgment of the investigators according to the SmPC. Principal investigators at each site had at least 5 years of expertise in administering systemic anticancer treatments. We defined duration of treatment as time from the date of the first dose of atezolizumab plus bevacizumab to the date of treatment discontinuation. OS was defined as the time from the date of the first dose of the treatment to the date of death. PFS was defined as the time from the date of the first dose of the treatment to the date of death or the date of radiological evidence of tumor progression. Time‐to‐progression (TTP) was defined as the time from the date of the first dose of the treatment to the date of radiological evidence of tumor progression. ORR was considered as the sum of the rates of CR and partial response (PR), assessed per RECIST criteria v1.1, whereas disease control rate (DCR) included the rates of CR, PR, and stable disease (SD). Radiological response and radiological diagnosis of progression were assessed locally by experienced radiologists in each center, without any central imaging review.

### Statistical analysis

We used descriptive statistics to summarize demographics. We used Fisher’s exact test or χ2 test to compare nominal, as appropriate. OS and PFS curves were calculated using the Kaplan‐Meier method. All statistical analysis were carried out with IBM SPSS Statistics version 28.0, MedCalc version 19.1.3, and GraphPad Prism version 8.0.2.

### Ethical considerations

The study was conducted according to the ethics guidelines in the Declaration of Helsinki. Ethical approval to conduct this study was granted following review of the study protocol by the Imperial College Tissue Bank (Reference Number R16008) and locally by the ethical committee of each participating site. Informed consent was not considered necessary by the review committee due to the retrospective nature of the study.

## RESULTS

### Patient characteristics

**FIGURE 1:**
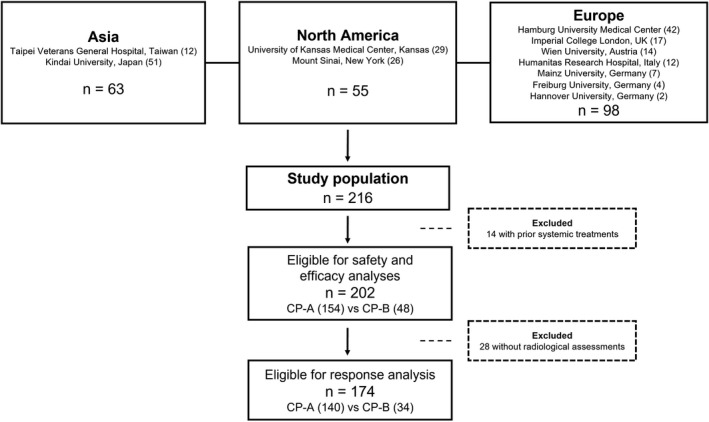
Flowchart of the study

We enrolled 216 patients treated consecutively with atezolizumab plus bevacizumab (Figure 1). We considered eligible for the analysis only patients receiving the combination as first‐line systemic treatment (*n* = 202, 94%). Median age was 69 years (range 23–90), with 85% of patients being male. The most frequent underlying liver disease was chronic viral hepatitis, secondary to HCV (*n* = 72, 36%) or HBV infection (*n* = 35, 17%). Most of the patients had a clinical or radiological diagnosis of cirrhosis (80%) and median time from initial diagnosis of HCC to start of atezolizumab plus bevacizumab was 6.7 months (interquartile range [IQR] 2.8–10.8). At treatment commencement, 127 patients (63%) were of Eastern Cooperative Oncology Group performance status (ECOG‐PS) 0. As shown in Table 1, 153 patients (71%) had received at least one prior locoregional or radical treatment, and the most frequent prior therapy was transarterial chemoembolization (27%).

**TABLE 1:** Clinical characteristics of the patients in the overall population

Characteristics	Data set *n* = 202 (%)
Age in years	
Median (range)	69 (23–90)
Sex	
Male	173 (85)
Female	29 (15)
Cirrhosis	
Absent	41 (20)
Present	161 (80)
Risk factor for liver disease	
HCV infection	72 (36)
HBV infection	35 (17)
HCV and HBV coinfection	3 (1)
Nonviral etiology	92 (46)
*Alcohol*	*39*
*NASH*	*23*
*Cryptogenic*	*30*
CP Class	
A	154 (76)
*A5*	*96*
*A6*	*58*
B	48 (24)
*B7*	*21*
*B8*	*21*
*B9*	6
ALBI grade	
1	71 (35)
2	118 (59)
3	13 (6)
BCLC Stage	
A	3 (2)
B	55 (27)
C	144 (71)
ECOG‐PS	
0	127 (63)
1	70 (35)
2	5 (2)
PVT	
Absent	122 (60)
Present	80 (40)
EHS	
Absent	125 (62)
Present	77 (38)
PVT and/or EHS	131 (65)
PVT and EHS	27 (13)
**Baseline AFP** ≥ 400 ng/ml	65 (32)
Prior locoregional treatment for HCC	
No prior treatment	62 (31)
Resection	51 (25)
Ablation	41 (20)
Transarterial chemoembolization	54 (27)
Transarterial radioembolization	20 (10)
External beam radiotherapy	6 (3)

Abbreviation: AFP, alpha‐fetoprotein.

The majority of patients were staged as C according to BCLC criteria (144 patients, 71%). Eighty patients (40%) had evidence of PVT and 77 (38%) of extrahepatic spread (EHS) at baseline, mostly at one metastatic site (*n* = 56). Twenty‐seven patients (13%) had evidence of both PVT and EHS. Baseline liver function was assessed according to CP score and albumin‐bilirubin (ALBI) grade: 154 patients (76%) were in CP‐A functional class and 48 (24%) were CP‐B, including 21 B7, 21 B8, and 6 B9. When categorized according to ALBI grade, 71 patients (35%) were graded as ALBI 1, 118 (59%) ALBI 2, and 13 (6%) ALBI 3. Baseline alpha‐fetoprotein value was ≥400 ng/ml in 65 patients (32%).

Considering the 161 patients diagnosed with cirrhosis, 31% of them (*n* = 50) had ascites and 12% (*n* = 19) encephalopathy. Compared with the general population, patients with cirrhosis had a similar percentage of PVT (40%, *n* = 65), ECOG‐PS score of 0 (63%, *n* = 102), CP‐B (27%, *n* = 44), ALBI 2 (60%, *n* = 96), and 3 (8%, *n* = 13).

### Safety

All patients who received at least one dose of atezolizumab plus bevacizumab were monitored for the development of treatment‐related AEs (trAEs). Median follow‐up time was 9.0 months (95% CI, 7.8–10.1).

A total of 143 patients (71%) suffered from any grade trAEs, of which 53 (26%) experienced a grade 3 trAE and 3 (1%) grade 4 trAE. Twenty‐five patients (12%) reported a grade ≥3 atezolizumab‐related AE, whereas 31 (14%) reported a grade ≥3 bevacizumab‐related AE (Table 2). The three grade 4 trAEs of the cohort were an atezolizumab‐related diarrhea, a bevacizumab‐related mesenterial venous thrombosis, and a bevacizumab‐related bleeding event from esophageal varices. Treatment‐related toxicity led to treatment discontinuation in 11 patients (5%), namely for three bevacizumab‐related AE (three bleeding events from gastroesophageal varices) and eight atezolizumab‐related AE (three colitis, and one each for pneumonitis, nephritis, fatigue, hepatotoxicity, thyrotoxicosis). Atezolizumab‐related AEs required steroids administration in 15 patients (7%), namely for hepatitis (*n* = 5), skin toxicity (*n* = 4), diarrhea (*n* = 4), and pulmonary and neurologic toxicity (*n* = 1 each).

**TABLE 2:** Treatment‐related AEs occurring during the treatment in the safety population

	Atezolizumab plus Bevacizumab (*n* = 202)	
Any grade trAEs relating to either drug (%)	143 (71%)	
Grade≥3 trAEs relating to either drug (%)		
*Atezolizumab‐related AE*	56 (28%)	
Grade 3	24 (12%)	
Grade 4	1 (1%)^a^	
*Bevacizumab‐related AE*		
Grade 3	29 (14%)	
Grade 4	2 (1%)^a^	
trAEs leading to treatment discontinuation relating to either drug (%)	11 (5%)	
Atezolizumab‐related AE requiring steroids	15 (7%)	
trAEs atezolizumab‐related (%)	Any grade	Grade ≥ 3
Overall	95 (47%)	24 (12%)
Fatigue	37 (18%)	2 (1%)
Hepatotoxicity	28 (14%)	12 (6%)
Colitis	26 (13%)	7 (3%)
Skin toxicity	19 (9%)	0
Thyroid toxicity	9 (4%)	1 (1%)
Arthritis	4 (2%)	1 (1%)
Pneumonitis	3 (1%)	2 (1%)
Neuropathy	1 (1%)	1 (1%)
Nephritis	1(1%)	0
trAE bevacizumab‐related (%)	Any grade	Grade ≥ 3
Overall	91 (45%)	31 (15%)
Hypertension	47 (23%)	9 (4%)
Proteinuria	41 (20%)	9 (4%)
Bleeding	28 (14%)	12 (6%)
Thrombosis	11 (5%)	5 (2%)

^a^ The three grade 4 trAEs of the cohort were an atezolizumab‐related diarrhea, a bevacizumab‐related mesenterial venous thrombosis, and a bevacizumab‐related bleeding event from esophageal varices.

Data about pretreatment esophagogastroduodenoscopy (EGD) were available for 108 patients (53%), with a median of 17 days from EGD to treatment start (IQR 1–137). In total, 63 patients of those who underwent a pretreatment EGD had evidence of gastroesophageal varices (58%), graded as 1 (*n* = 33), 2 (*n* = 18), and 3 (*n* = 12), respectively. Varices were managed according to local practice, with either banding or medical treatment. Among the patients who received a baseline EGD, 41 (38%) received prophylactic treatment. In particular, 14 (13%) underwent band ligation, 15 (14%) were on beta‐blockers, and 12 (11%) received both banding and pharmacological treatment. Of the remaining 67 patients (62%) without any prophylactic treatment, 45 (42%) did not have baseline varices, and 22 (20%) had grade 1 varices that did not need a specific treatment. Prophylactic treatment was administered in 65% of patients with a variceal finding, whereas the remaining 35% untreated patients had all grade 1 varices.

**FIGURE 2:**
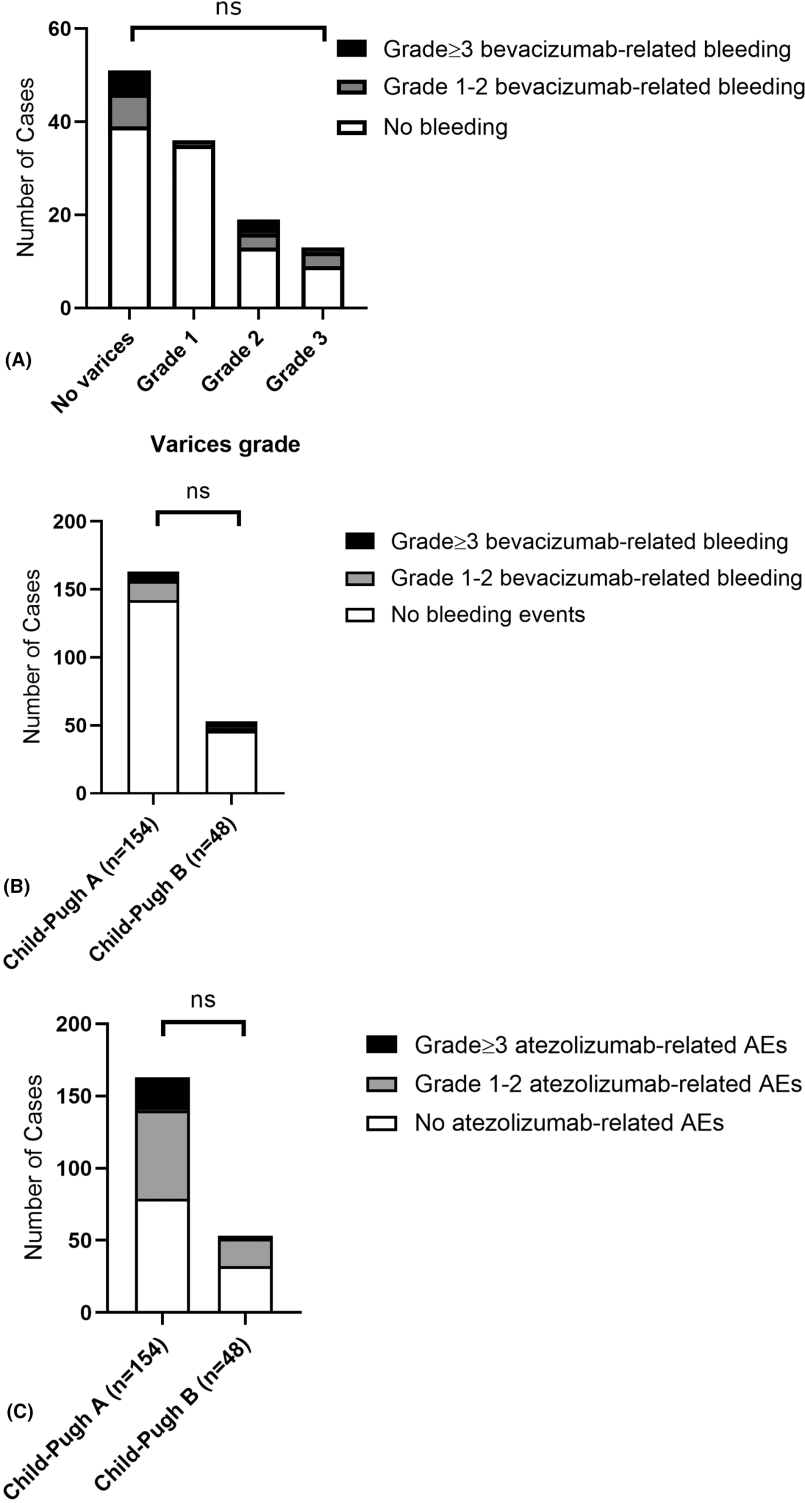
Graphical representation of (A) the number of GI bleeding events in relationship with grade of varices at pretreatment EGD; (B) the number of bevacizumab‐related bleeding events across CP classes; (C) the number of atezolizumab‐related AEs across CP classes. [Correction statement added May 16, 2022 after first online publication: the values in the x‐axis were corrected in Figures 2B and 2C]

The rate of bleeding events in the safety population was 14% (*n* = 28), of whom 8% (*n* = 16) were of grade 1–2, 5% (*n* = 11) of grade 3, and 1% (*n* = 1) of grade 4 as per CTCAE v5.0 criteria. The grade ≥3 bleeding events included nine cases of gastroesophageal variceal bleeding (one grade 4) and one case each of epistaxis, HCC rupture, and duodenal ulcer bleeding. Bleeding events were not associated with BCLC stage, CP class, ALBI grade, or administration of prophylactic treatment for varices (*p* > 0.05 for all associations). Also, we did not find any association between the presence of baseline PVT and development of bleeding events of any grade: 13 patients suffered from bleeding in the group without PVT (*n tot* = 122) and 15 in the group with PVT (*n tot* = 80; *p* = 0.10). The presence of varices at the pretreatment EGD did not correlate with the development of gastrointestinal (GI) bleeding events of any grade, which were reported by 12 patients among the 51 without varices at EGD (24%) and by 11 patients among the 68 with varices (16%; *p* = 0.31, Figure 2A).

The development of grade ≥3 atezolizumab‐related and bevacizumab‐related AEs was not influenced by the underlying etiology (viral vs. not viral), baseline PVT, baseline ECOG‐PS, BCLC stage, baseline ALBI grade (*p* > 0.05 for all associations). When comparing patients with CP‐A and patients with CP‐B in terms of toxicity, no difference was observed (Figure 2B,C). In particular, bevacizumab‐related AEs of any grade were reported by 74 patients with CP‐A (48%) and by 22 patients with CP‐B (46%), whereas grade ≥3 bevacizumab‐related AEs were developed by 24 patients with CP‐A (16%) and 7 patients with CP‐B (15%). The proportion of GI bleeding events was not dissimilar in patients with CP‐A versus CP‐B when considering events of any grade (14% vs. 15%) and grade ≥3 (4% vs. 10%). Bleeding events in patients with CP‐B were not associated with baseline PVT, ECOG‐PS, sex, etiology (viral vs. not viral), BCLC stage, or presence of cirrhosis (*p* > 0.05 for all associations). Eighty‐two patients with CP‐A (53%) and 19 patients with CP‐B (40%) complained of any grade atezolizumab‐related AEs, whereas grade ≥3 atezolizumab‐related AEs were reported by 23 patients with CP‐A (15%) and 2 patients with CP‐B (4%). Twenty‐three patients with CP‐A (15%) and 5 (10%) patients with CP‐B suffered from any grade atezolizumab‐related hepatitis with 12 cases of grade ≥3 hepatitis in the CP‐A group (8%) and none in the CP‐B group (*p* > 0.05 for all associations).

### Efficacy

**FIGURE 3:**
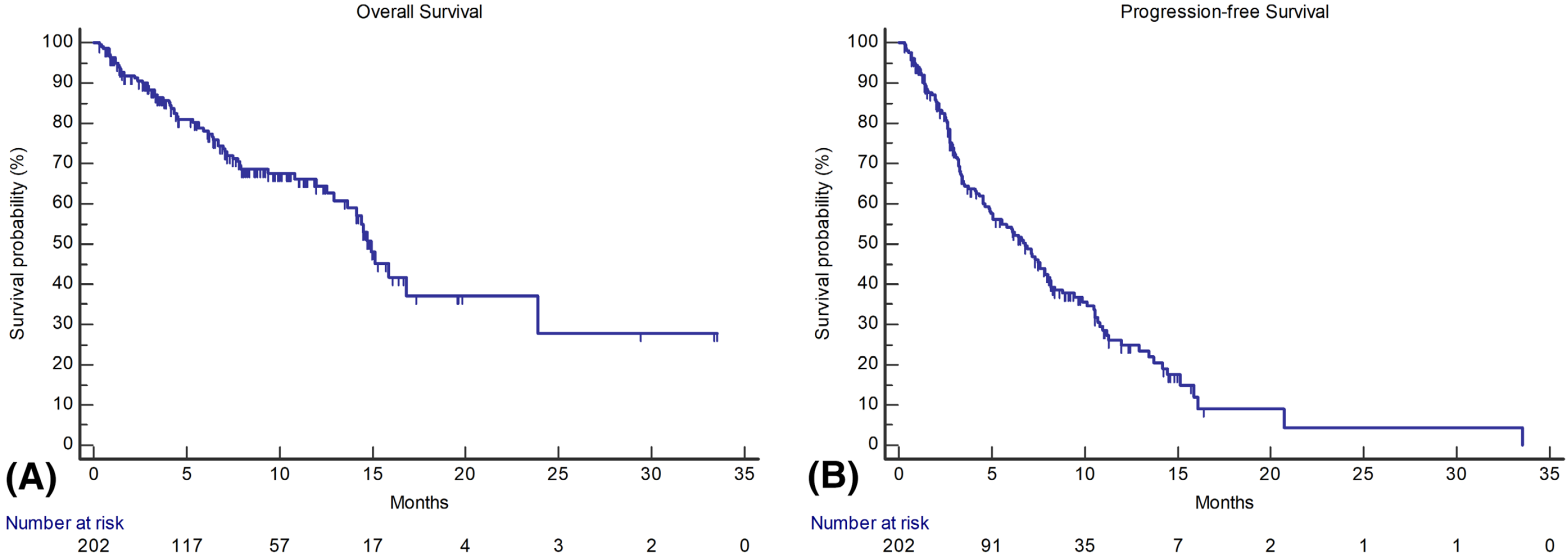
Kaplan‐Meier curves describing the OS (A) and the PFS (B) of the efficacy population, including patients treated in first line only

**FIGURE 4:**
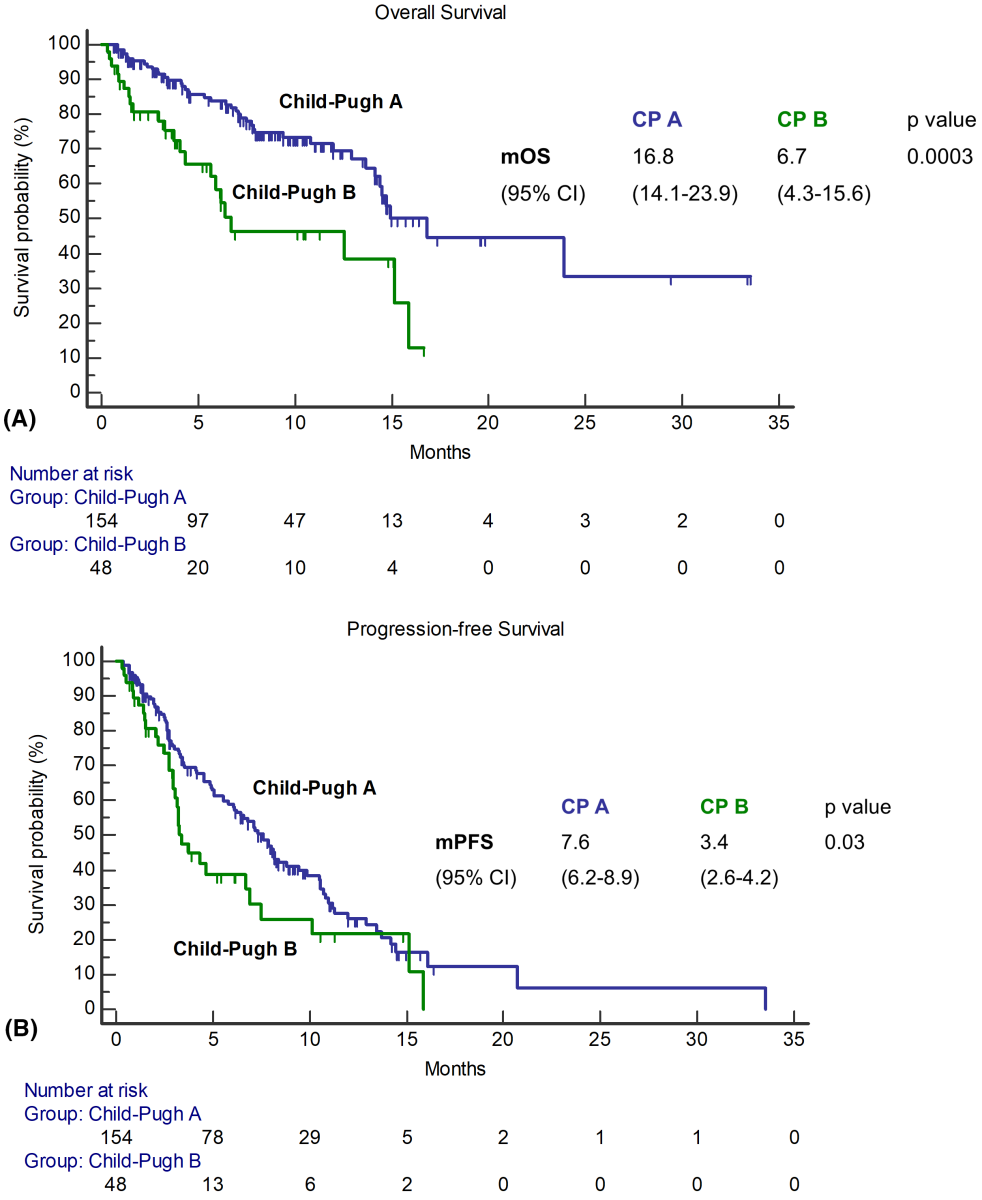
Kaplan‐Meier curves describing the OS (A) and the PFS (B) of the efficacy population stratified per CP class

In the overall population, mOS was 14.9 months (95% CI, 13.6–16.3; Figure 3A). The 6‐month survival rate was 77%, whereas the 12‐month survival rate was 60%. Patients with CP‐A achieved an mOS of 16.8 months (95% CI, 14.1–23.9), whereas it was 6.7 months (95% CI, 4.3–15.6) for patients with CP‐B (*p* = 0.0003) (Figure 4A). The mPFS of the overall sampled population was 6.8 months (95% CI, 5.2–8.5; Figure 3B), whereas it was 7.6 months (95% CI, 6.2–8.9) for patients with CP‐A and 3.4 months (95% CI, 2.6–4.2) for patients with CP‐B (*p* = 0.03) (Figure 4B). The whole cohort achieved a median TTP of 7.2 months (95% CI, 5.8–8.5). When comparing across CP classes, we found that patients with CP‐A reached a median TTP of 7.6 months (95% CI, 6.4–8.8) versus 4.6 months (95% CI, 0.8–8.4) in patients with CP‐B (log rank *p* = 0.28). Radiological response was assessed in 174 patients (86%) according to RECIST v1.1 criteria. Among these patients, one (1%) achieved a CR, 42 (24%) a PR, and 84 (48%) an SD, whereas progressive disease was the best response for 47 (27%) patients. ORR was 25%, and the DCR was 73% (Table 3). Response was comparable across CP classes, with ORR being 26% in CP‐A and 21% in CP‐B, and it was not influenced by BCLC staging, ECOG‐PS, etiology (viral vs. nonviral), PVT, or EHS (*p* > 0.05 for all associations).

**TABLE 3:** Best radiological response evaluated per RECIST criteria version 1.1

Atezolizumab plus Bevacizumab	Overall Population (*n* = 174^a^)	CP‐A (*n* = 140)	CP‐B (*n* = 34)
ORR^b^, %	25%	26%	21%
DCR^c^, %	73%	74%	68%
CR	1%	1%	0
PR	24%	25%	21%
SD	48%	48%	47%
Progressive disease	27%	26%	32%

^a^ Radiological response was available for 174 patients (86% of the efficacy population).

^b^ ORR was considered as the sum of complete and partial responses.

^c^ DCR was considered as the sum of complete and partial responses and SD.

Median treatment duration was 3.5 months (IQR 1.9–8.0), and it was longer in patients with CP‐A liver function compared with CP‐B class (4.3 vs. 2.9, *p* = 0.029). At data cutoff in January 2022, 134 patients (66%) had discontinued treatment; 66 patients (33%) because of radiologically proven disease progression, 27 (13%) because of clinical deterioration, 16 (8%) because of death, 12 (6%) because of unacceptable toxicity, and 13 (6%) for other reasons.

When radiological progression was assessed, most of the patients (49%) suffered from intrahepatic progression, whereas 24% experienced an extrahepatic progression, and 28% suffered from combined intrahepatic and extrahepatic progression. After treatment discontinuation, 57 patients (43%) received a further line of systemic treatment, of whom 39 received a tyrosine kinase inhibitor (24 lenvatinib, 5 sorafenib, 2 cabozantinib, 8 unknown) and 18 received another immune checkpoint inhibitor‐based treatment.

## DISCUSSION

Combination immunotherapy has reshaped the treatment landscape of liver cancer, providing a standard of care characterized by increased long‐term efficacy compared with other treatment options for unresectable HCC.^[^ ^21^ ^]^ The uptake of this therapeutic option has not been comprehensively assessed outside clinical trials.

In this multicenter study, we evaluated the experience in the use of atezolizumab and bevacizumab in routine clinical practice, focusing on safety and efficacy outcomes. By interrogating a prospectively maintained global registry of patients treated with immunotherapy,^[^ ^13^, ^15^, ^16^, ^17^ ^]^ we were able to confirm that the combination of atezolizumab plus bevacizumab is a safe and effective option also when administered in a real‐life setting.

We systematically collected data regarding the trAEs that occurred during treatment. Atezolizumab plus bevacizumab was confirmed to be a tolerable treatment. In our study, there was no evidence of treatment‐related deaths, nor evidence of newly emerging safety signals compared with clinical trial data sets. In particular, the rate of patients discontinuing treatment due to trAEs was strikingly similar between our study and IMbrave150 (5% and 7%, respectively).^[^ ^6^ ^]^ Because the risk of GI bleeding is a key concern in assessing candidacy to atezolizumab and bevacizumab, we sought to evaluate whether presence and endoscopic grade of esophageal varices were associated with bleeding events. There is a proportion of patients who may suffer adverse outcomes, and this has been identified as patients with Vp4 (presence of a tumor thrombus in the main trunk and/or contralateral portal vein).^[^ ^22^ ^]^ However, although limited by retrospective design and by the lack of available data for a part of the population, our study shows no correlation between the presence of varices at the pretreatment EGD and the development of treatment‐related bleeding events for patients who underwent a baseline EGD assessment, showing that systematic screening and timely introduction of prophylaxis are effective in preventing bleeding events. The microvascular changes induced by bevacizumab can spark serious complications when used on the background of portal hypertension,^[^ ^23^ ^]^ and in a historical case series from phase II trials, where the variceal prophylaxis was less standardized, bevacizumab‐related bleeding events occurred in up to 10% of the patients.^[^ ^24^ ^]^ Our findings further corroborate the importance of routine EGD assessment before treatment, which was mandated per protocol within the IMbrave150 trial,^[^ ^6^ ^]^ and it has to be considered compulsory as part of good routine practice. If identified and adequately treated, the presence of varices is not associated with the risk of GI bleeding, thus making the delivery of atezolizumab plus bevacizumab a safe option in this population. Reasons for incomplete adherence to pretreatment EGD screening are impossible to fully reconstruct in retrospective studies. However, a significant proportion of patients received treatment during the COVID‐19 pandemic, when access to EGD was deprioritized in view of the high risk of mortality in patients with cancer.^[^ ^25^ ^]^

Another important finding of our study is the description of the use of atezolizumab plus bevacizumab in patients with CP‐B liver dysfunction, which accounted for almost one third of the study population. Decision to treat patients with CP‐B was made in the context of multidisciplinary consensus among participating institutions as a likely consequence of the unprecedented landmark mOS endpoint of 19.2 months reached by patients treated with this combination in IMbrave150,^[^ ^7^ ^]^ the favorable impact on quality of life of the combination^[^ ^26^ ^]^ and evolving knowledge in the field, suggesting that PD‐1 monotherapy is safe in patients with CP‐B liver impairment.^[^ ^12^, ^13^, ^14^, ^16^ ^]^

Treatment with atezolizumab and bevacizumab in patients with CP‐B was well tolerated, with no relevant difference in terms of clinically significant trAEs attributable to either drug in comparison with patients with CP‐A.

Evidence of good tolerability of the combination is of particular importance given the risk of potentially life‐threatening bleeding events secondary to bevacizumab and the potential for atezolizumab to further compromise liver dysfunction by triggering immune‐related hepatitis. Reassuringly, the proportion of patients who experienced bleeding events in our study was comparable between patients with CP‐A and patients with CP‐B, not diverging from rates observed in IMbrave150. Similarly, rates of severe atezolizumab‐related AEs were not dissimilar across groups, even when hepatic events of clinical interests were considered.

Furthermore, we conducted an exploratory efficacy analysis. When compared with the IMbrave150 updated results,^[^ ^7^ ^]^ the mOS of our population appeared to be numerically shorter (14.9 vs. 19.2 months in our population and in the IMbrave150 study, respectively), whereas mPFS was comparable (6.8 vs. 6.9 months). The difference in terms of OS is not surprising given the shorter follow‐up interval of our study compared with the updated analysis (9.0 vs. 15.6 months, respectively),^[^ ^7^ ^]^ with 34% of patients still receiving treatment. However, the median follow‐up of our study is comparable with the follow‐up of the original publication of the IMbrave150 study, which was 8.6 months for the whole cohort.^[^ ^6^ ^]^ The presence of patients clustering within CP‐B functional reserve and patients with ECOG‐PS 2 are also variables with a predicted impact on survival estimates. Despite the documented differences in the patient populations, our study confirms the efficacy of atezolizumab plus bevacizumab in terms of ORR measured by RECIST v1.1 criteria, with a reported estimate of 25% in our study comparable with the 27.3% seen in IMbrave150.^[^ ^6^ ^]^ Although patients in CP‐B functional class were characterized by a worse survival compared with patients with CP‐A in view of the competing effect of liver dysfunction over tumor progression on patients’ mortality, reassuringly, response rates did not seem to differ across different CP functional classes. Furthermore, to minimize the concurrent risk of death due to the underlying liver impairment, we measured the median TTP across CP classes. Unlike the PFS, the measure of the TTP excludes death events from the time‐to‐event analyses, as it takes into account only radiological progression. We found that, coherently with the absence of significant differences in terms of radiological response rate, atezolizumab plus bevacizumab achieved comparable median TTP in patients with CP‐A and B liver function.

Taken together, our findings suggest that the combination of atezolizumab plus bevacizumab may be safely administered even beyond the strict inclusion criteria of the IMbrave150 study. Treatment of patients with a mildly impaired liver function represents a major unmet need for the hepato‐oncology community^[^ ^27^ ^]^ because large, randomized phase III clinical trials have traditionally excluded patients with CP‐B liver function.^[^ ^28^ ^]^ However, CP‐B is a heterogeneous subset of patients with HCC, encompassing varying degrees of hepatic impairment. As shown in previous experience with sorafenib therapy,^[^ ^29^ ^]^ careful patient selection is key to safely consider systemic therapy even in this more fragile population. To our knowledge, the only immunotherapy treatment prospectively tested for safety and efficacy in patients with CP‐B is nivolumab.^[^ ^12^ ^]^ Considering that mAbs do not undergo hepatic metabolism^[^ ^30^ ^]^ and that their pharmacokinetics does not imply a dose adjustment in patients with a mildly impaired liver function,^[^ ^31^ ^]^ our data are provocative in suggesting prospective testing of atezolizumab plus bevacizumab in a selected subgroup of patients with CP‐B. However, given the retrospective nature of our study and the relatively small sample size, these findings should be regarded as purely hypothesis‐generating, warranting evaluation in prospective, adequately powered clinical trials.

A number of different immune checkpoint inhibitor‐based treatment strategies are currently under investigation in global randomized phase III clinical trials,^[^ ^10^, ^11^, ^32^, ^33^ ^]^ and the upcoming results are likely to enrich the first‐line treatment landscape. Also, evidence regarding the sequencing of different tyrosine kinase inhibitors shows competitive results in terms of survival,^[^ ^34^, ^35^, ^36^ ^]^ making the choice of first‐line treatment particularly challenging for the categories of patients excluded from clinical trials. For this reason, studies providing real‐life data are precious to disentangle the treatment algorithm and to provide further evidence for patient stratification.

Our study acknowledges a number of important limitations. In the first instance, the retrospective nature of the data set, albeit prospectively maintained, cannot substitute level I evidence from prospective studies. This study should be regarded as mainly safety‐oriented, and the efficacy findings should be considered only exploratory, including the shorter OS compared with the updated IMbrave150 results.^[^ ^7^ ^]^ However, the comparable outcome in terms of mPFS highlighted the reproducibility of the results in real life. In this light, our preliminary findings on use of atezolizumab and bevacizumab beyond first line and in patients with CP‐B should be interpreted with caution as they are not meant to change clinical practice. In particular, our study enrolled 53 patients within CP‐B class, and therefore it was not powered to evaluate outcomes across the individual CP scores 7–9, a point that should be explored in future studies. Global phase IV and local phase IIIb programs are underway to prospectively validate safety and efficacy of atezolizumab and bevacizumab in real‐world patient cohorts, whereas other phase II prospective trials are investigating the use of the combination exclusively in patients with CP‐B (AB7 ‐ NCT04829383 in the United States; CHALLENGE ‐ jRCTs031210355 in Japan). Similar to our study, results from the sorafenib era published in the GIDEON study, highlight an unsurprising divergence between clinical trial and postregistration evidence, with documented utilization of systemic therapy in patients where no clear survival benefit exists (i.e., CP‐B or BCLC D HCC).^[^ ^37^ ^]^

Also, the real‐life setting of our study implies a lack of standardization in clinical practice including eligibility assessment, frequency of follow‐up, and management of AEs. Real‐world studies including the present one need to address the issue of missing data. Lack of EGD data in part of the population, for instance, might have led to selection bias. Lastly, considering the relatively small sample size, our results should be considered speculative, especially for what concerns the identification of prognostic factors. Despite the acknowledged limitations, our study confirms that the combination of atezolizumab plus bevacizumab is tolerable and effective in patients with unresectable HCC who are treated in routine clinical practice. Lack of correlation between endoscopic severity of esophageal varices and bleeding events provides clinically useful data to guide the decision‐making process in clinical practice. Patients with CP‐B cirrhosis deserve prospective evaluation of safety efficacy of this combination in dedicated clinical studies.

## CONFLICT OF INTEREST

AD received educational support for congress attendance from Roche. JvF received advisory board fees from Roche. HW received lecture fees and advisory board honoraria from Roche, Bayer, Ipsen, Eisai, BMS. VEG is employee and shareholder of F. Hoffmann‐La Roche, Ltd. AS received research grants (to institution) from AstraZeneca, Merck, Bristol Myers Squibb, Exelixis, Clovis, KAHR medical, Actuate therapeutics, Incyte Corp. and Advisory board fees from AstraZeneca, Bristol Myers Squibb, Merck, Exelixis, and Pfizer. PRG reports a consulting or advisory role and received honoraria from AdaptImmune, AstraZeneca, Bayer, Bristol Myers Squibb, Eisai, Ipsen, Lilly, Merck Sharp & Dohme, Roche, and Sirtex; has been on a speakers bureau for straZeneca, Bayer, Bristol Myers Squibb, Eisai, Ipsen, Lilly, Merck Sharp & Dohme, Roche, and Sirtex; has received research funding from Bayer and Roche; has provided expert testimony for Lilly; and has received travel or accommodation expenses from AstraZeneca, Bayer, Bristol Myers Squibb, Eisai, Ipsen, Lilly, and Roche. DB has received lecture and speaker fees from Bayer Healthcare, the Falk Foundation Germany and consulting fees from Boston Scientific. AV reports honoraria for speaker, consultancy and advisory role from Roche, AstraZeneca, EISAI, Bayer, Merck, Bristol Myers Squibb, Merck Sharp & Dohme, Incyte, PierreFabre, Ipsen, and Sanofi. BS received travel support from Gilead, Ipsen and AbbVie. NP received consulting fees from Amgen, Merck Serono, Servier; lectures fees from AbbVie, Gilead, Lilly, Sanofi; travel expenses from Amgen, ArQule; and institutional research funding from Basilea, Merck Serono, Servier. TP received consulting fees from Bayer; and institutional research funding from Bayer, Lilly, Roche. RS received consulting fees for EISAI, Roche, Bayer, SIRTEX, Novartis; research funding (to institution) from Incyte, Novartis, Astex Pharmaceuticals, Bayer and Boston Scientific. MP is an investigator for Bayer, BMS, Ipsen, Lilly, and Roche; he received speaker honoraria fromBayer, BMS, Eisai, Lilly, MSD, and Roche; he is a consultant for Bayer, BMS, Eisai, Ipsen, Lilly, MSD, and Roche; he received travel support from Bayer and BMS. AC received consulting fees from MSD, BMS, AstraZeneca, Roche; speakers’ fee from AstraZeneca, MSD, Novartis and Astellas. LR received consulting fees from Amgen, ArQule, AstraZeneca, Basilea, Bayer, BMS, Celgene, Eisai, Exelixis, Genenta, Hengrui, Incyte, Ipsen, IQVIA, Lilly, MSD, Nerviano Medical Sciences, Roche, Sanofi, Servier, Taiho Oncology, Zymeworks; lecture fees from AbbVie, Amgen, Bayer, Eisai, Gilead, Incyte, Ipsen, Lilly, Merck Serono, Roche, Sanofi; travel expenses from Ipsen; and institutional research funding from Agios, ARMO BioSciences, AstraZeneca, BeiGene, Eisai, Exelixis, Fibrogen, Incyte, Ipsen, Lilly, MSD, Nerviano Medical Sciences, Roche, Zymeworks.DJP received lecture fees from ViiV Healthcare, Bayer Healthcare, BMS, Roche, Eisai, Falk Foundation, travel expenses from BMS and Bayer Healthcare; consulting fees for Mina Therapeutics, EISAI, Roche, DaVolterra, Mursla, Exact Sciences and Astra Zeneca; research funding (to institution) from MSD and BMS. All remaining authors have declared no conflicts of interest. The authors have no other relevant affiliations or financial involvement with any organization or entity with a financial interest in or financial conflict with the subject matter or materials discussed in the manuscript apart from those disclosed. No writing assistance was utilized in the production of this manuscript.

## AUTHOR CONTRIBUTIONS

*All the authors contributed to writing and revising the original draft. Antonio D’Alessio contributed to data curation, formal analysis, conceptualization, and visualization; David J. Pinato and Lorenza Rimassa contributed to supervision and project administration*.

## Data Availability

The data that support the findings of this study are available on request from the corresponding author. The data are not publicly available due to privacy or ethical restrictions.
